# Census-derived migration data as a tool for informing malaria elimination policy

**DOI:** 10.1186/s12936-016-1315-5

**Published:** 2016-05-11

**Authors:** Nick W. Ruktanonchai, Darlene Bhavnani, Alessandro Sorichetta, Linus Bengtsson, Keith H. Carter, Roberto C. Córdoba, Arnaud Le Menach, Xin Lu, Erik Wetter, Elisabeth zu Erbach-Schoenberg, Andrew J. Tatem

**Affiliations:** WorldPop, Geography and Environment, University of Southampton, Southampton, SO17 1BJ UK; Flowminder Foundation, Stockholm, Sweden; Clinton Health Access Initiative, Boston, MA USA; Karolinska Institute, Stockholm, Sweden; Pan American Health Organization/World Health Organization, Washington, DC USA; Department of Health Surveillance, Costa Rica Ministry of Health, San Jose, Costa Rica; Stockholm School of Economics, Stockholm, Sweden; Fogarty International Center, National Institutes of Health, Bethesda, MD USA

**Keywords:** Malaria elimination, Human mobility, Census data, Migration, Mobile phone data

## Abstract

**Background:**

Numerous countries around the world are approaching malaria elimination. Until global eradication is achieved, countries that successfully eliminate the disease will contend with parasite reintroduction through international movement of infected people. Human-mediated parasite mobility is also important within countries near elimination, as it drives parasite flows that affect disease transmission on a subnational scale.

**Methods:**

Movement patterns exhibited in census-based migration data are compared with patterns exhibited in a mobile phone data set from Haiti to quantify how well migration data predict short-term movement patterns. Because short-term movement data were unavailable for Mesoamerica, a logistic regression model fit to migration data from three countries in Mesoamerica is used to predict flows of infected people between subnational administrative units throughout the region.

**Results:**

Population flows predicted using census-based migration data correlated strongly with mobile phone-derived movements when used as a measure of relative connectivity. Relative population flows are therefore predicted using census data across Mesoamerica, informing the areas that are likely exporters and importers of infected people. Relative population flows are used to identify community structure, useful for coordinating interventions and elimination efforts to minimize importation risk. Finally, the ability of census microdata inform future intervention planning is discussed in a country-specific setting using Costa Rica as an example.

**Conclusions:**

These results show long-term migration data can effectively predict the relative flows of infected people to direct malaria elimination policy, a particularly relevant result because migration data are generally easier to obtain than short-term movement data such as mobile phone records. Further, predicted relative flows highlight policy-relevant population dynamics, such as major exporters across the region, and Nicaragua and Costa Rica’s strong connection by movement of infected people, suggesting close coordination of their elimination efforts. Country-specific applications are discussed as well, such as predicting areas at relatively high risk of importation, which could inform surveillance and treatment strategies.

**Electronic supplementary material:**

The online version of this article (doi:10.1186/s12936-016-1315-5) contains supplementary material, which is available to authorized users.

## Background

Though malaria remains a global health priority and causes an estimated 438,000 deaths annually [1], mortality has declined dramatically in recent decades [2, 3] and several countries around the world are approaching parasite elimination. Country-specific elimination is an important step towards the ultimate goal of malaria eradication [4], and requires both stopping transmission within national borders and management of imported malaria. Importation and within-country transmission dynamics depend greatly upon human movement patterns, as human-mediated parasite mobility facilitates source-sink dynamics within a country and drives importation risk from international exporters of infected people [5, 6]. Thus, malaria control programmes should take into account human movement and malaria mobility when designing malaria elimination plans to achieve elimination in a robust and efficient way [7].

Finding appropriate human movement information for predicting malaria mobility is difficult, as movement varies in duration, frequency, and spatial scale [8, 9], and reliable, globally-consistent movement data are difficult to obtain [10]. Various movement typologies are captured by different methods, each with inherent advantages and disadvantages [11]. For example, short-term circulatory movement can be captured using mobile phone call data records, which document the towers that rout a user’s calls and texts. By observing the locations of towers utilized by a user over time, short-term movement patterns can be inferred to yield important insights into local disease dynamics [12–14]. Often, these data do not record cross-border movements, however [10], as network operators generally only provide service within a single country. Future mobile phone data could reflect international movement if they include roaming calls/texts or handset identifiers which could be used to link users between network operators, but most currently available mobile phone data are restricted to a single country.

Census data and other migration-oriented data such as migrant stock data can begin to fill these gaps [10], as censuses often include questionnaires regarding previous residence or birthplace including international origins [11]. Further, these data are more readily available than mobile phone records [11], making them applicable for a larger number of countries. Spatial connectivity between subnational regions can be gleaned from these migration-oriented data by analysing population flows between pairs of subnational administrative units, and models fit using these data can be used to predict flows between administrative units in the same country [11, 16] and in different countries [17]. Their direct utility for predicting malaria parasite flows is limited, however, as they record longer-term migration related movements, a minor component of overall parasite mobility [18].

Ideally, regional mapping of malaria connectivity should integrate the strengths of short-term movement data (such mobile phone records, travel history surveys, or GPS tracking) with more readily available data sets such as census-based migration. Importantly, previous studies suggest that migration and mobile phone data exhibit similar general patterns that are robust across spatial scales of movement [19]. Though census migration data greatly under predict flows compared to mobile phone call data records, using relative instead of absolute flows yields similar connectivity networks in both data sets. Because census-based migration data are readily available and typically more representative of the population at-large than mobile phone data, these data can be used across large scales to predict relative flows and connectivity maps. Validation against data sets that capture more frequent movements is necessary to ensure predictive accuracy for predicting malaria parasite movement [19], however.

This study first confirms whether data reflect similar general patterns as short-term movement data by comparing migration patterns in census microdata with movement in a mobile phone data set from Haiti. The mobile phone and census data sets complement each other reasonably well for this validation exercise, as mobile phone data capture short-term movement effectively even in the context of demographic biases in mobile phone ownership [20] and migration data capture the international movements necessary for regional mobility mapping. The migration data are then used to predict relative flows of infected people between first-level administrative units throughout Mesoamerica, with accompanying discussion on how these flows can guide policy design. A final discussion focuses on Costa Rica to show the utility of these measures in directing country-specific elimination policy.

Mesoamerica is an important setting for these analyses, as countries throughout Mesoamerica are rapidly approaching elimination with overall declines of >9 % each year from 2000 to 2011 in annual parasite incidence, or the number of cases appearing at health facility per year, per 100,000 individuals [21]. Elimination efforts will be enhanced by appropriately accounting for human mobility across national borders [22], especially as Mesoamerica exhibits the highest emigration rates in the world [23] and has highly mobile migrant labour populations [24]. Significant regional programmatic support for elimination has been provided through programmes such as RAVREDA/AMI and the Mesoamerican Health Initiative 2015 as well [25], and the presented analyses can help elucidate regional parasite movement to guide these programmes into the future.

## Methods

First, movement patterns in mobile phone and census microdata from Haiti are compared to validate whether the census data can predict short-term movement. Logistic regression models fit using both data sets are compared to determine if movement patterns differed with respect to covariates known to be good predictors of subnational movement [16]. Then, a similarly structured model is fit using census microdata from El Salavdor, Costa Rica, and Nicaragua, used to predict population flows throughout Mesoamerica. Combined with regional incidence estimates, model results are used to predict regional flows of infected people, community membership, net export, and net import of infected people for each administrative unit. Finally, because Costa Rica census data were available for these analyses, country-specific analyses are shown to discuss how they can specifically inform national policy.

All statistical analyses in this manuscript are performed in R version 3.1.1 [26] and the glm and lmer functions from the lme4 package [27]. The data frames of output data and administrative unit metadata are provided in Additional files 2 and 3. In these files, the “uidfr” and “uidto” variables in the output data frame link with “uid” in the provided table of administrative unit names and codes. These administrative unit names and code correspond to the Food and Agriculture Organization Global Administrative Unit Layers (GAUL), which are available from the FAO website [28]. Further, Table 1 contains the fitted model coefficients, which can be used to generate predictions for other regions.

**Table 1:** Coefficients for best fit logistic regression model using census microdata from El Salvador, Costa Rica, and Nicaragua

	Estimate	Standard error	Z score
Log (population at origin)	0.0098	0.010	0.98
Log (population at destination)	0.906	0.0098	92.81***
Log (distance between centroids)	−0.306	0.011	−26.67***
Contiguity	0.878	0.014	62.90***
Proportion of population in urban areas, origin	−0.221	0.038	−5.84***
Proportion of population in urban areas, destination	0.379	0.038	10.05***

* Indicates statistical significance at p ≤ 0.05, ** Indicates significance at p ≤ 0.01, and *** Indicates significance at p < 0.001

### Data sources and model specification

#### Census microdata

The census microdata originate from the Integrated Public Use Microdata Series, International (IPUMSI; [29]). The validation exercise uses mobile phone data from 2010 and census microdata from a census of Haiti in 2003. In a separate modelling exercise, migration data from censuses of El Salvador (2007), Costa Rica (2011), and Nicaragua (2005) are used to predict population flows between pairs of admin units across Mesoamerica. These data sets are subnationally representative census subsets, recording the first level administrative unit that individuals currently reside in, as well as their home first level administrative unit 5 years in the past if within the same country. These data are freely available online [29]. These data are used to calculate the proportion of residents 5 years prior to the census who moved to each other unit, for each administrative unit, as an indicator of relative short-term population flow. Using proportions rather than actual flows avoids applying a model fit to the 5–10 % subsample that the microdata represent to the much larger population sizes of the entire population.

#### Mobile phone call record data

The mobile phone data set was provided by Digicel, the largest GSM mobile phone network operator in Haiti with 90 % coverage of inhabited areas across the country [12]. These data consist of anonymized data on all SIM cards that made at least one call, and record the last tower utilized by each user for each day between September 1, 2010 and December 1, 2010. The data included 2.2 million subscribers (SIM cards) over the study period, during which 171 million call/text events were recorded. Therefore, there was an average of 59 days with call/text data for each SIM during the 90-day study period. Movement patterns extracted from the data have previously been shown to correspond closely to movement patterns reported during the same period in a large-scale representative household survey [30], suggesting that demographic biases in mobile phone ownership have a limited effect on observed movement patterns.

By comparing the locations of towers that routed a call or text with locations of towers used for the subsequent call or text over all users for the study period, this analysis calculates the proportion of individuals near one tower who transitioned to another per call/text event. Because the presented models use proportions of people who moved between geographical units rather than actual flows, it was not necessary to account for discrepancies in apparent population sizes caused by biased mobile phone ownership. Other biases may affect observed patterns, however, such as spatial biases in call rates, which could affect apparent proportions of people who moved. Further analysis of this mobile phone data set and its possible biases is available in Additional file 1.

### Logistic regression models

The presented logistic regression models predict proportions of people from geographical unit *i* who moved to another *j* per time step. This proportion is calculated as the number of people who moved from *i* to *j* in a given data set, $mig_{i,j}$, divided by the total number of people in the sample recorded within *i*, $tot_{i}$.

The included covariates are distance between administrative unit centroids ($dist_{i,j}$), whether or not the administrative unit pair is contiguous ($contig_{i,j}$; a binary variable), total population ($pop_{i}$) and the proportion of people in urbanized areas ($urbprop_{i}$) for both the origin and destination administrative units. Total population was obtained from the WorldPop Project [31, 32], and population rasters for 2010 were combined with an urbanization layer [33] to obtain the proportion of people in urbanized areas. These covariates are included because of their ability to predict intranational migratory movements across numerous countries [16]. The basic model is shown below:

$$\frac{{mig_{i,j} }}{{tot_{i} }} = \beta_{0} \,+\, \beta_{1} { \log }(pop_{i} ) \,+\, \beta_{2} { \log }(pop_{j} ) \,+\, \beta_{3} urbprop_{i} \,+\, \beta_{4} urbprop_{j}\, +\, \beta_{5} dist_{i,j} \,+\, \beta_{6} contig_{i,j}$$

where $\beta_{i}$ indicates the coefficient for the covariate *i*. This model includes log of population size because population sizes are skewed with a few administrative units containing a large proportion of the population.

### Haiti comparison

Movement patterns are initially compared between census microdata from Haiti with mobile phone data. Because the census data recorded movements between second-level administrative units (arrondissements), cell towers in the mobile phone data are aggregated to their respective arrondissements. Three arrondissements (out of 42) did not contain any mobile phone towers, and so excluded from these analyses.

From the mobile phone data, population flows between arrondissements ($mig_{i,j}$) is the number of times individuals utilized a tower in an arrondissement and then subsequently used a tower in another, regardless of the time that elapsed between the two calls. A corresponding $mig_{i,j}$ value in the census microdata is calculated as the number of people who lived in an arrondissement 5 years ago and had moved to another by the time of the census. Ranked flows are compared between admin units rather than the actual values because migration data categorically underpredict short-term movement patterns [19].

Logistic regression models are also fit using both data sets, and comparing the directionality and magnitude of fitted coefficients ensures that movement is similar with respect to the covariates used throughout this study. To calculate $\frac{{mig_{i,j} }}{{tot_{i} }}$ in the mobile phone data set, the total effective population for an arrondissement *i* ($tot_{i}$) is defined as the total number of days across all SIMs where the last recorded tower was in *i*. Ultimately, then, the proportion $\frac{{mig_{i,j} }}{{tot_{i} }}$ corresponding to each arrondissement pair indicates the probability that a SIM in *i* subsequently moved to *j* by the following call/text event. In the census data, the corresponding proportions $\frac{{mig_{i,j} }}{{tot_{i} }}$ is defined by dividing the number of people in arrondissement *i* that lived in another arrondissement *j* 5 years ago ($mig_{i,j}$) by the total population originally assigned to *i* 5 years ago ($tot_{i}$), reflecting probabilities of individuals moving on average.

As probabilities of an individual moving between geographical units is the outcome of interest in both the mobile phone data and the census data, these outcomes differ only in time period. For the mobile phone data, the relevant period for the transition probabilities is the average duration between call/text events, roughly 1.52 days, and for the census data, this period is 5 years.

### Mesoamerica movement

Census data from El Salvador (2007), Costa Rica (2011), and Nicaragua (2005) are used to fit a logistic regression model that predicted connectivity across Mesoamerica. Only data on subnational movement was available, as the census data did not record origin first-level administrative unit for international migrants. The model is identical to the model fit using the Haiti data, except it included country-level random effects during fitting to account for national differences in movement. Only the fixed effects are used to predict proportions of people that moved per 5 years between all possible first-level admin unit pairs (both within and between countries) across Mesoamerica. Using the proportions of people predicted to move between administrative units, population flows are obtained by multiplying proportion with the total population in the origin admin unit (generated by summing a population raster obtained from the WorldPop project [31, 32] per administrative unit).

Applying this model to administrative unit pairs in different countries assumes that country borders are completely porous, as the model is fit using only subnational migration. As this is an unrealistic assumption, predicted international migratory movements are scaled using an existing data set on predicted international migration [15]. This data set comprehensively predicts crossborder migration between all countries nationally on the same timescale as the census microdata (per 5 years). While bilateral migration flows can be difficult to obtain using census information as statistical agencies do not necessarily collect migration data in a comparable way, this data set is predicted using population stock data, which are more widely available and easier to measure across countries [15]. This adjustment rescales all movements from one country to another such that net flow between the countries matched the international predictions. Therefore, the results assume that while relative patterns of international and subnational movement are identical in the context of model covariates, international movement is considerably rarer than subnational movement.

To predict the relative flows of people infected with malaria (either *Plasmodium falciparum* or *Plasmodium vivax*), predicted population flows are scaled using estimates of malaria incidence across Mesoamerica from a data set provided by the Pan American Health Organization. These data record the number of people diagnosed with either *Pf* or *Pv* malaria per month at health facilities across Mesoamerica for 2013, aggregated to second-level administrative units. Annual incidence estimates at the appropriate spatial scale are calculated by averaging incidence across each first-level admin unit in a population-weighted manner using population estimates from the WorldPop Project [32].

Predicted flows of infected people is then the product of these first-level administrative unit incidence estimates and predicted population flows. This relationship between incidence and flow of infected people assumes that transmission intensity in an area correlates linearly with the proportion of emigrants that are infected, which is reasonable in low transmission settings when multiple infection is rare [34] such as Mesoamerica.

### Community structure

After predicting flows of infected individuals across Mesoamerica, groups of admin units most closely linked are defined using a walktrap community detection algorithm [35]. This algorithm iteratively places random walkers at various administrative units, and the probability of a walker moving to other administrative unit depends on predicted flow between those administrative units. This algorithm is able to utilize weighted graphs (or edges with associated non-binary values, such as the predicted flow values in this example), but requires a symmetrized adjacency matrix, therefore defined between admin unit *i* and *j* as predicted total flow ($mig_{i,j}$) between the two units in either direction.

Over many iterations, random walkers will tend to travel to administrative units within the same community, and this algorithm defines subcommunity membership in a “bottom-up” way using random walker locations. All administrative units are initially in independent subcommunities, and the two subcommunities most often visited by the same random walker are merged iteratively until the difference between movement within and between subcommunities is maximized (represented by a modularity score, $Q$ [35, 36]). By maximizing the difference in movement when comparing between and within subcommunity movement, this algorithm defines the best partitioning of administrative units across the region.

The resulting partitioning then represents groups of administrative units (or subcommunities) that infected people are most likely to pass between during travel. Coordination of effort across administrative units in the same subcommunity minimizes importation risk, as coordination can prevent areas lacking active intervention effort from becoming sources of infected people for the subcommunity at-large.

### Overall movement of infected people

Flows of infected people are also used to define major exporters and importers of infected people. Quantifying exportation and importation rates can help target elimination efforts, as net exporters are disproportionately important for overall malaria persistence [37]. As exportation and importation rates are defined independently, a unit can be both a top exporter and importer. An administrative unit’s relative role as an exporter or importer is then the net number of infected people expected to leave or enter each administrative unit per 5 years through migration-related movement, ranked against all other administrative units. Presenting exportation and importation rate ranks as opposed to the absolute values of predicted exportation and importation accounts for the significant underprediction of short-term movement in flows predicted using migratory flows over 5 years.

Figure 4 shows the overall probability of an individual moving from each administrative unit, showing possible individual-level movement rather than population-level predictions of overall flow.

### Costa Rica migration

The final country-specific discussion demonstrates how these analyses can inform malaria elimination policy in a particular country. Census data from Costa Rica in 2011 (obtained from the Instituto Nacional de Estadística y Censos) are used for these analyses rather than the IPUMSI data to map movement in Costa Rica. The census data set has more detailed information on the origin of international migrants (though this information was still at the country-level rather than first-level administrative unit-level) and apply countrywide without necessitating an underlying logistic regression model. Relative levels of imported malaria expected to reach each province are estimated using these flows.

## Results and discussion

### Haiti comparison and limitations

**Fig. 1:**
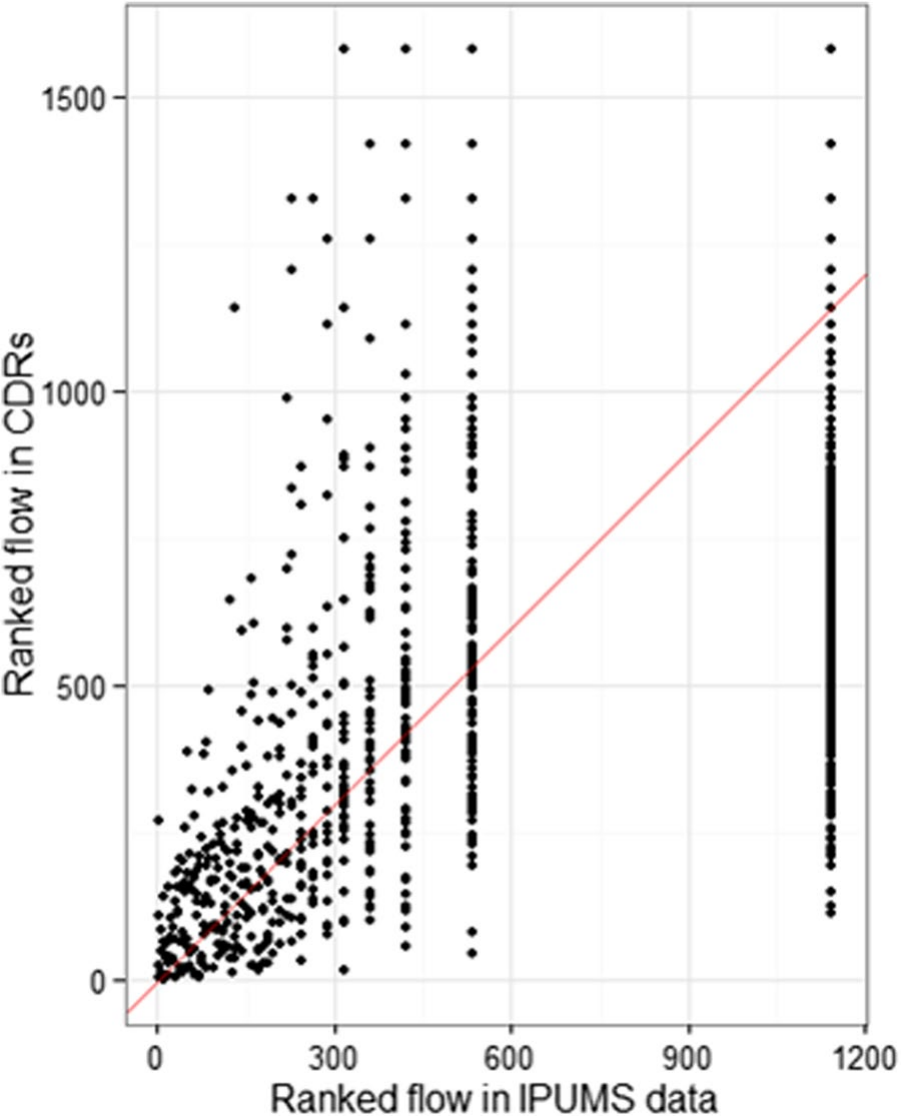
Ranked pairwise movement in the census microdata and the mobile phone data. Calculated R^2^ between these rankings was 0.69. The IPUMS microdata originate from Haiti in 2003, while the mobile phone data originate from Haiti in 2010. Observations falling at rank 1150 in the IPUMS data represent pairs where no migration occurred

**Fig. 2:**
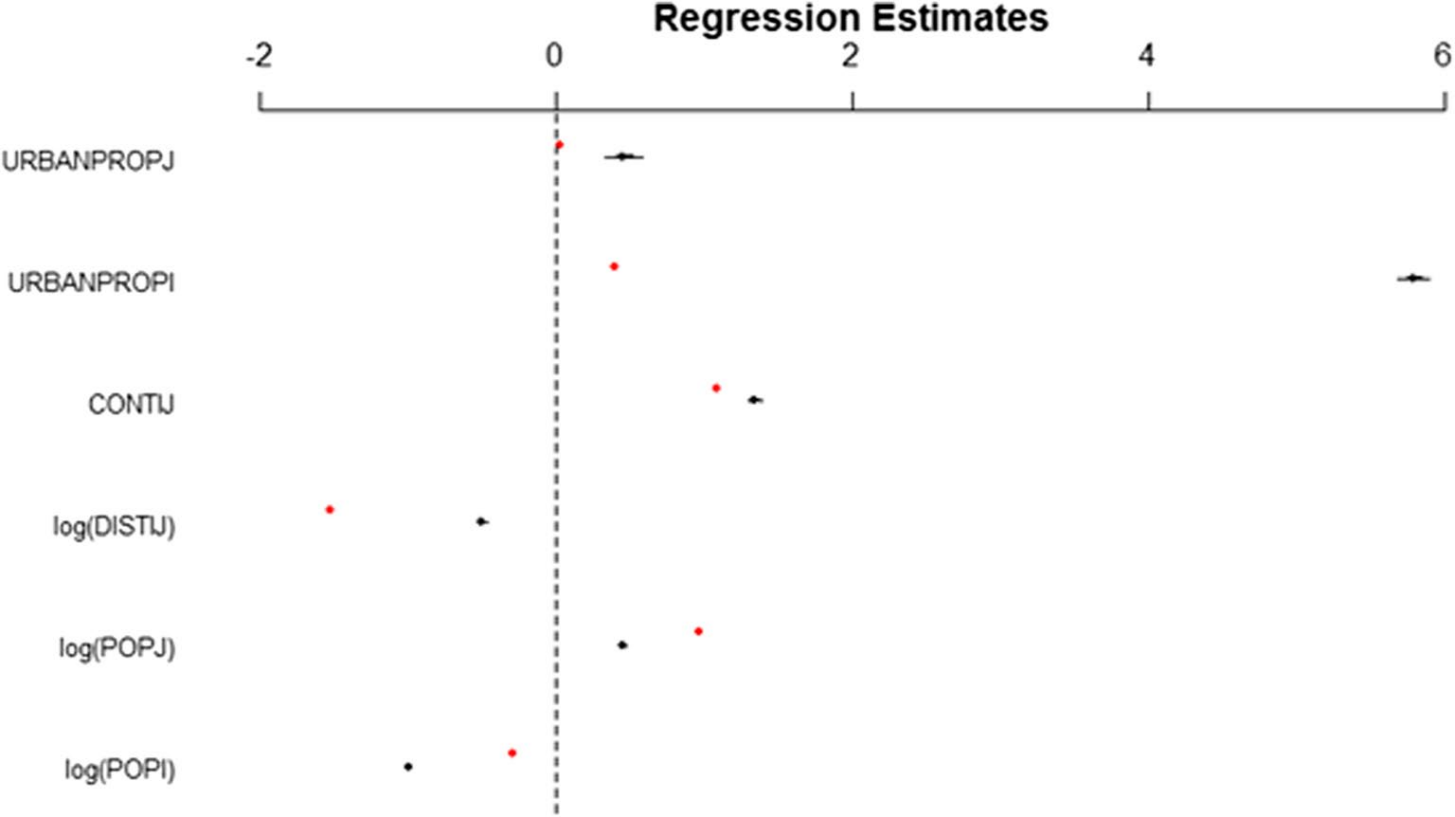
Logistic regression model coefficients after fitting with mobile phone and IPUMS census microdata. *Red dots* indicate coefficients from mobile phone data-derived model, and *black* indicate coefficients from census-derived model with corresponding 95 % confidence intervals

Ranked flows between all pairs of districts in Haiti correlate well between the mobile phone and the census data ($R^{2} = 0.69$; Fig. 1), a stronger correlation than observed in previous studies that compared mobile phone and census data [19]. The logistic regression model also yields similar results when fit to both data sets (Fig. 2), suggesting it predicts circulatory movement patterns well with respect to the model covariates used.

Though this result suggests that the census microdata exhibit similar patterns to the mobile phone data, the mobile phone data do not necessarily represent a complete, unbiased picture of short-term movement. Mobile phone ownership is known to be demographically biased, and while movement patterns have been shown to be robust to income-based biases in the data [20], certain populations such as undocumented migrants or roaming international travellers may not be represented in these data. Further, biases may still exist in the calculated transition probabilities and flows due to more or less frequent mobile phone use during travel, which has been documented previously [38]. Additional file 1 contains analyses regarding some of these possible biases.

### Mesoamerica movement

Migration in the census microdata is significantly positively correlated with urbanization in the destination administrative unit, population size in the destination, and negatively correlated with distance and urbanization in the origin admin unit (Table 1), implying that people tend to move to closer, more highly populated, and highly urbanized areas, while tending not to leave highly urbanized areas. These results are generally similar to the Haiti models (Fig. 2), though these models indicate a higher probability of moving out of highly urbanized areas, rather than lower, as in the Mesoamerica census microdata). This difference may reflect unusually high rates of movement out of Port-au-Prince in Haiti in the aftermath of the 2010 earthquake captured in the CDR data [30], or may reflect spatial biases in the CDR data. This overall similarity between the Haiti and Mesoamerica models is relevant for applicability of the validation exercise. Had the models differed dramatically, the validation of movement throughout Haiti may not have applied to the fundamentally different patterns found in Mesoamerica. Though the models were broadly similar, this remains a possible concern, and mobile phone data from Mesoamerica should be used in the future to validate the Mesoamerican census data-derived movement patterns.

**Fig. 3:**
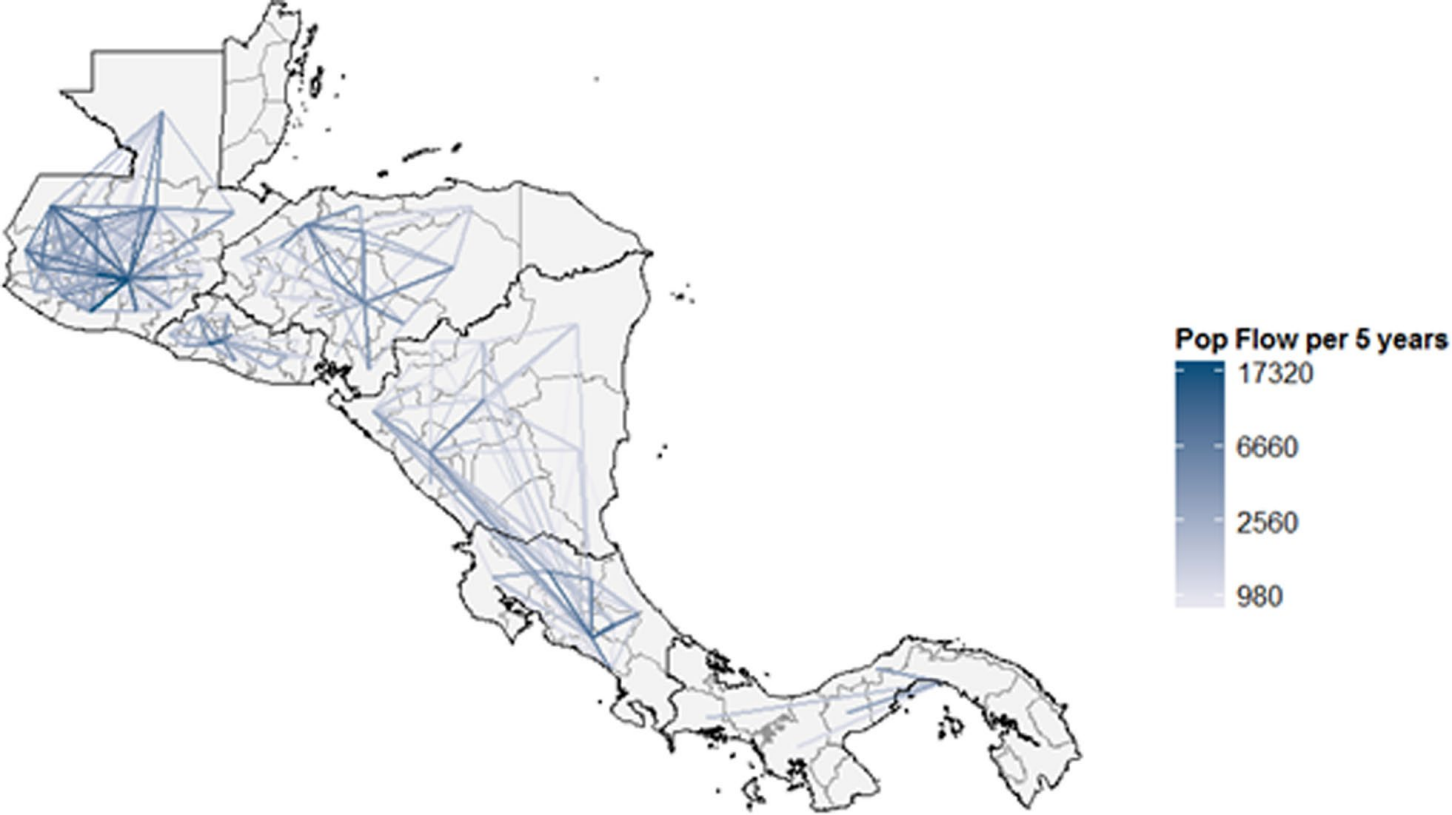
Predicted migratory flow (per 5 years) between first-level administrative units across Mesoamerica. These population flows are generated from a logistic regression model fit using census data and scaled using crossborder predictions from Abel and Sander [15]

**Fig. 4:**
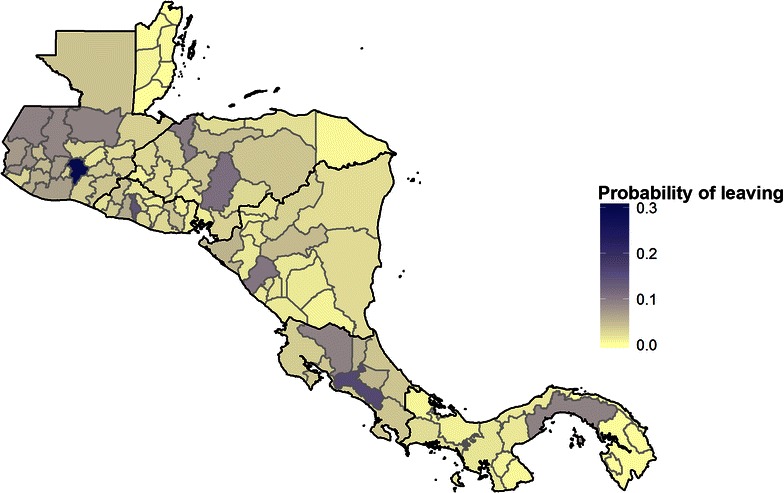
Overall predicted probability of a resident leaving each administrative unit over 5 years. Crossborder probabilities scaled using Abel and Sander [15]

Figure 3 shows regional movement across Mesoamerica obtained once scaled using data from Abel and Sander [15], while Fig. 4 shows the probabilities that underlie these predictions, as the net probability of leaving each administrative unit over 5 years. The probabilities shown in Fig. 4 include the crossborder scaling, accomplished by dividing flows by the total population in the origin administrative unit to yield scaled probabilities.

Figure 4 shows probability of leaving an administrative unit $i$, calculated as one minus the probability of not leaving to go to all other possible administrative units, or $1 - \prod\nolimits_{j = 1,i \ne j}^{n} {(1 - p_{i,j} )}$ where $p_{i,j}$ is the predicted probability of travelling from $i$ to $j$. Rather than reflecting predicted net flows, then, Fig. 4 identifies the administrative units where infected people might be most likely to travel elsewhere, carrying infection with them.

**Fig. 5:**
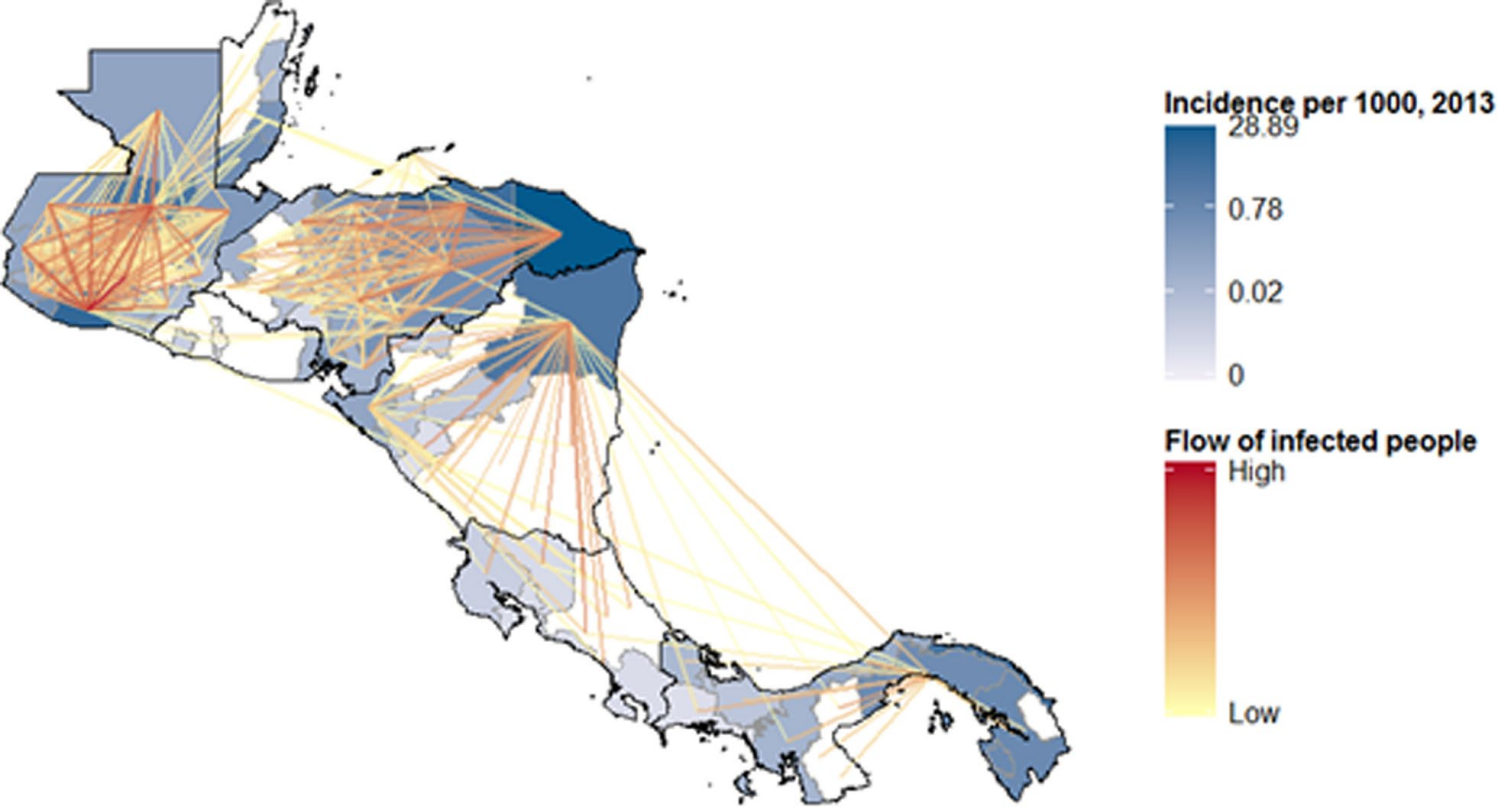
Predicted flows of infected people (*red*). These estimates are created using population flow estimates from Fig. 1 and scaling using incidence from 2013 in the origin location (shown in *blue*)

**Fig. 6:**
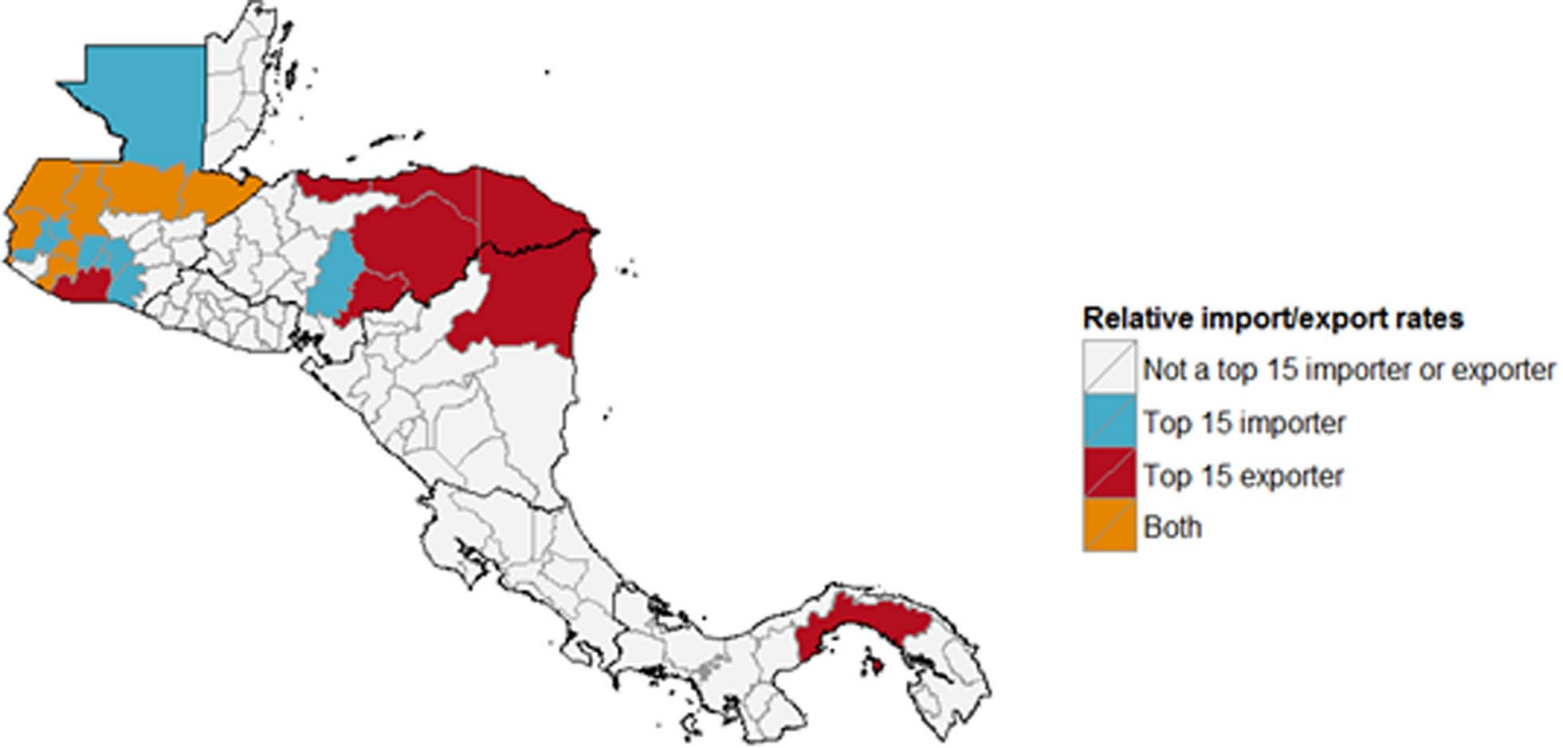
Top 15 exporters and importers of malaria-infected individuals throughout the region

Combining Fig. 3 with PAHO incidence data, Fig. 5 depicts net flows of infected people and Fig. 6 shows areas that act as major exporters or importers of infected people. This visualization is particularly policy-relevant, as reducing transmission in major exporters of infected people is likely to reduce burden in other areas. Transmission reduction in exporting areas can be achieved by targeting mosquito populations, through interventions such as vector control and insecticide-treated net distributions, or through interventions that target the infectious reservoir in humans, including active case detection and strengthened treatment programmes. Areas that are both major exporters and importers are also important targets, as they act as conduits of infected people, and would particularly benefit from active detection of infection in travellers.

**Fig. 7:**
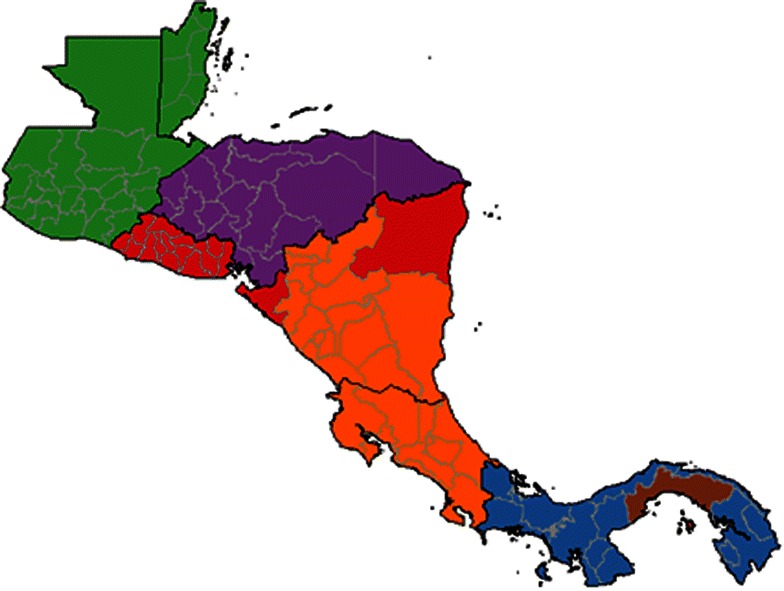
Community structure of infected people throughout Mesoamerica. Community structure is defined using a walktrap community detection algorithm. *Colours* denote administrative units belonging to the same subcommunity

Figure 7 shows optimal partitioning of the region into geographic subcommunities using these flows, showing that while most countries are relatively isolated and form independent subcommunities, Nicaragua and Costa Rica share a subcommunity, as do Belize and Guatemala. Taken alone, this analysis emphasizes international coordination of elimination efforts along particular national boundaries. The shared community membership of Nicaragua and Costa Rica, for example, suggests that if Costa Rica reduces transmission below replacement levels within its borders, malaria may persist due to importation from Nicaragua. Combined with Figs. 5 and 6, this analysis highlights northern Nicaragua as a particularly important exporter of infected people across multiple countries, as relatively many people are expected to flow into Costa Rica from this area (darker red lines; Fig. 5), and it also likely is a major exporter of cases into El Salvador (Fig. 7).

### Costa Rica migration

**Fig. 8:**
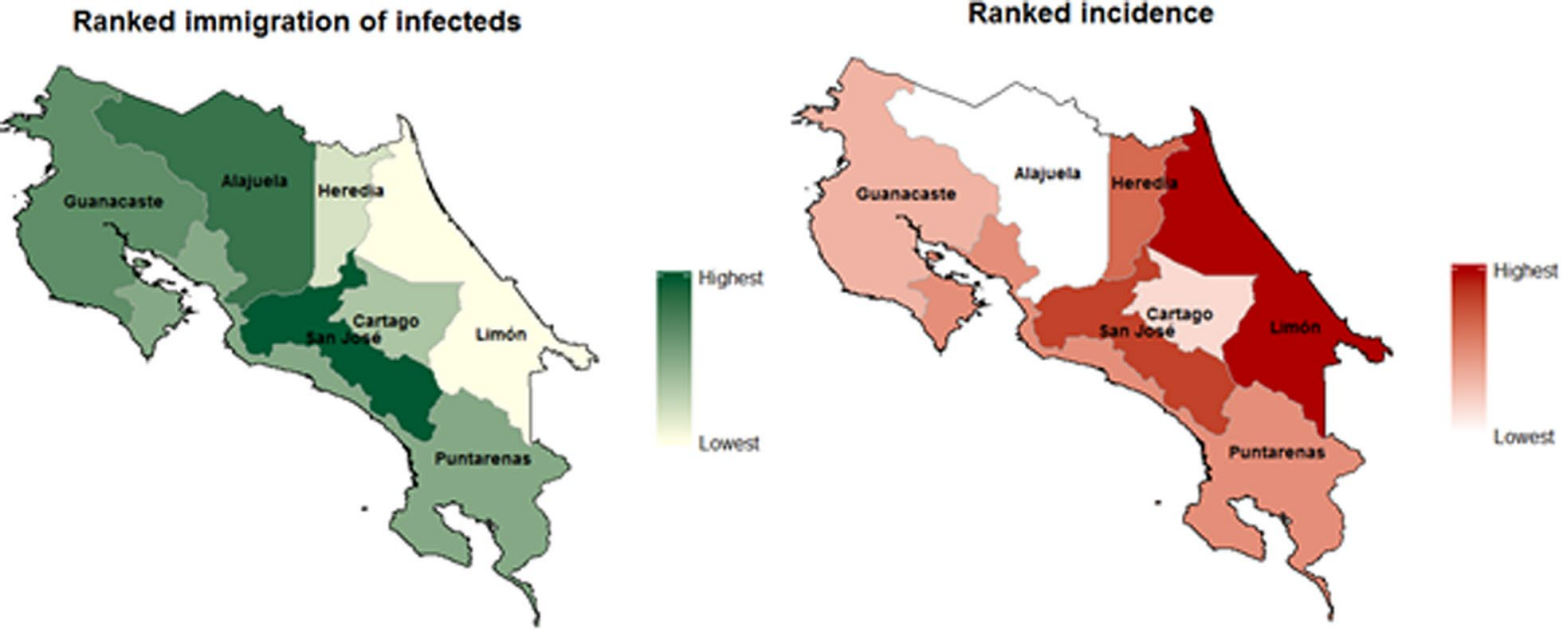
Migration and incidence throughout Costa Rica. *Left* Expected immigration of infected people into each province in Costa Rica. Migration rates are calculated by scaling migration from each origin with incidence in that origin, using PAHO incidence data from 2013 to define both intra- and international movement of infected people. *Right* Ranked incidence across Costa Rica, from PAHO data

Combining the Costa Rica census data with the PAHO incidence estimates, importation rates of infected people (from international and intranational sources) are calculated for province in Costa Rica. By comparing observed incidence across the country with the expected rates of importation, it is possible to determine the provinces most likely to sustain local transmission and the provinces likely to experience proportionally more importation. Figure 8 shows the expected relative rates of immigration of infected people against observed patterns of malaria burden. This is relevant for surveillance, as while Limon has the highest observed incidence, it receives very few infected migrants, implying that local transmission rather than importation may be the main source of infection. In contrast, Alajuela experienced relatively few cases from 2008 to 2010 but is expected to experience relatively high levels of immigration of infected people, suggesting that cases may be imported from elsewhere and that less transmission may be occurring within the province. These results justify extending malaria diagnosis and treatment to highly mobile populations (particularly undocumented migrants) in provinces where international-specific importation is high, and by justifying potential active case detection in travellers in areas with high overall importation risk.

### Census-derived model limitations

While this study agrees with previous work in showing that migration data are useful for predicting malaria parasite movement [19], using these data to model parasite mobility carries assumptions and limitations that should be addressed by future research. In particular, while the movement model is deliberately simple for generalization across Mesoamerica, this simplicity also means the model cannot capture complex patterns of human movement, and does not reflect individual-level heterogeneities observed between people that are critically important for malaria elimination [39]. Travellers being potentially at higher risk or lower risk of malaria further muddles the relationship between parasite prevalence in travellers and overall incidence. Because of these unaccounted heterogeneities, the export/import predictions are highly uncertain, relying instead on a linear relationship between incidence and proportion of travellers infected and travellers moving identically to the population at large. In particular, then, the lack of information on a possible correlation between movement and infection risk represents a key limitation common to both the mobile phone and census data, as neither data set records individual-level risk. Future studies mapping parasite mobility could account for this by using appropriate data, such as travel history surveys from health clinics to infer possible correlation in infection risk and movement. By modelling demographic-specific subgroups and understanding how demography influences disease-risk and movement, future work can refine these predictions.

Further, in modelling cross-border movement, this analysis assumes that cross-border movement patterns are identical to intranational movement (though are much rarer), which may not reflect actual differences in processes that drive international movement. While cross-border movement information was available in the census microdata, this information was only at the country level, and therefore did not provide any additional information for quantifying whether movement between first-level administrative units differed internationally and subnationally. Other studies suggest that international migratory patterns differ significantly from intranational migration [17], underscoring the importance of future work including country-specific international movement information. For Mesoamerica, the highly porous nature of national borders regionally [40] suggests that cross-border movement may be less restricted than in other regions, and therefore potentially more similar to intranational movement.

Even if these microdata captured more information on migrant origin, they may miss populations at-risk of malaria and possible mediators of parasite movement, such as highly mobile indigenous populations. Some such at-risk populations include those within the Mosquitia, an indigenously populated region, which includes a relatively porous border between Honduras and Nicaragua, or the Darien in Panama where Guna communities often migrate to and from Colombia [41]. More comprehensive household surveys and travel history surveys can account for this, as they often record both international and subnational movement in a spatially granular way and can be targeted to reach underserved populations. Future data obtained from mobile network operators could also inform international movement patterns, as international movement could be tracked using information such as handset identifier codes, which could link records from network operators in different countries.

Despite these uncertainties, maps of parasite flows and importation created using migration data will be important for malaria elimination efforts regionally and on a country-specific basis, as they succinctly present complex movement processes in a general, policy-relevant way. The metrics shown here can inform distinctly different aspects of elimination. For example, understanding areas that are exporters of infected people, such as northern Nicaragua and eastern Honduras, can help target intervention campaigns. If targeted treatment and vector control programmes reduce transmission in these areas, burden will decline across the larger landscape. On the other hand, drawing community structures can inform coordination efforts between areas, such as between Costa Rica and Nicaragua, to minimize reintroduction risk (Fig. 6), and can help predict where importation is most likely to occur. This inference can inform implementation of policies geared towards providing case management and diagnosis for highly mobile populations within the country and active case detection in travellers (Fig. 7). The results of this work can be used in other settings, as Additional files 2 and 3 contains the output data for public use, Table 1 contains the fitted model parameters, and the associated IPUMS census microdata are available upon request [29]. By guiding surveillance and intervention resources towards the areas where they are most useful, analyses similar to those presented in this study can help achieve malaria elimination in a cost-effective way.

## Conclusions

Interactions between mobile human populations and spatially heterogeneous landscapes of malaria transmission lead to complex spatiotemporal disease dynamics [8, 9]. These complex disease dynamics are important for elimination, as they drive importation and resurgence even in post-elimination settings [5, 6]. This study presents maps of parasite connectivity for Mesoamerica, predicted using data on incidence and human population movement.

The presented analyses show that census-derived movement patterns are a reasonable proxy for relative flows observed in short-term circulatory movement (Figs. 1, 2), matching existing research [19], though significant assumptions and uncertainties remain to be addressed by future research. Ultimately, malaria risk and burden are driven by both human movement and transmission through highly interactive processes [8]. Understanding how both impact parasite dynamics and flows will be a critical step for defining effective intervention packages in different areas and informing overall elimination strategy.

## Additional files

10.1186/s12936-016-1315-5 Supplementary analysis of mobile phone data. This analysis shows some possible biases in the mobile phone data from Haiti and discusses briefly their possible implications.
10.1186/s12936-016-1315-5 Predicted movement throughout Mesoamerica. This is the output from the fitted model for Mesoamerica. The “uidto” and “uidfr” variables match with the “uid” variable in Additional file 3. The lat/lon fr/to variables represent centroids of the origin and destination administrative units, while POPI/POPJ refer to total population in the administrative unit in 2010. URBANPROPI/J refer to the proportion of the population in urbanized areas per administrative unit. “CONTIJ” refers to whether the units are contiguous, and “DISTIJ” refers to the distance in kilometres between administrative unit centroids. “predprop” is the proportion of people expected to move from i to j, “predpropnum” is this proportion multiplied by POPI, while “adjprednum” is this number adjusted using Abel and Sander [15]. Finally, “adjpredprop” is this adjusted flow converted back to a proportion (calculated by dividing “adjprednum” by population in i), and “infmove” is the adjusted flow multiplied by incidence in i (“incidfr”).
10.1186/s12936-016-1315-5 First-level administrative unit metadata. This metadata matches the model output from Additional file 2 with the FAO GAUL shapefile. “uid” matches with “uidto” and “uidfr” in Additional file 2. ADM0_CODE, ADM0_NAME, ADM1_CODE, and ADM1_NAME match with the corresponding variables in the FAO GAUL shapefile for 2013. “TotPop” is the total population in each administrative unit, “UrbProp” is the proportion of people in urbanized areas, and “x” and “y” refer to the centroids for each administrative unit. “probleave” is the probability of someone leaving each administrative unit, shown in Figure 4.
