# Supplementary material for: Census-derived migration data as a tool for informing malaria elimination policy
Source: Malar J. 2016 May 11;15:273. doi: 10.1186/s12936-016-1315-5 (PMC4864939; doi:10.1186/s12936-016-1315-5)
Supplement: Supplementary file 1 — 10.1186/s12936-016-1315-5 Supplementary analysis of mobile phone data. This analysis shows some possible biases in the mobile phone data from Haiti and discusses briefly their possible implications. [file 12936_2016_1315_MOESM1_ESM.docx]

**Supplementary analysis of mobile phone data**

Recent research suggests that mobile phone data correlates strongly movement exhibited in large-scale household surveys [1] and generally correlates with movement at-large despite demographic biases in mobile phone ownership [2]. Biases remain in these data, however, particularly because call/text events do not occur randomly and may occur more often during travel [3]. Here we illustrate possible biases in the mobile phone data and how they could influence our results.

In this sample, there were 2,898,664 individual SIMs. For these 2.9 million SIMs there were 171 million days with calls/texts between September and November 2010, yielding call/text events every 1.52 days on average. The actual number of days between days varied between 0 and 88 (where the mobile phone data subset used in this study covered 90 days). Overall, most calls/texts were separated by very few days, as 92.7% of calls/text pairs had zero days between them, and 99% of events had 5 or fewer days between them (Figure S1).


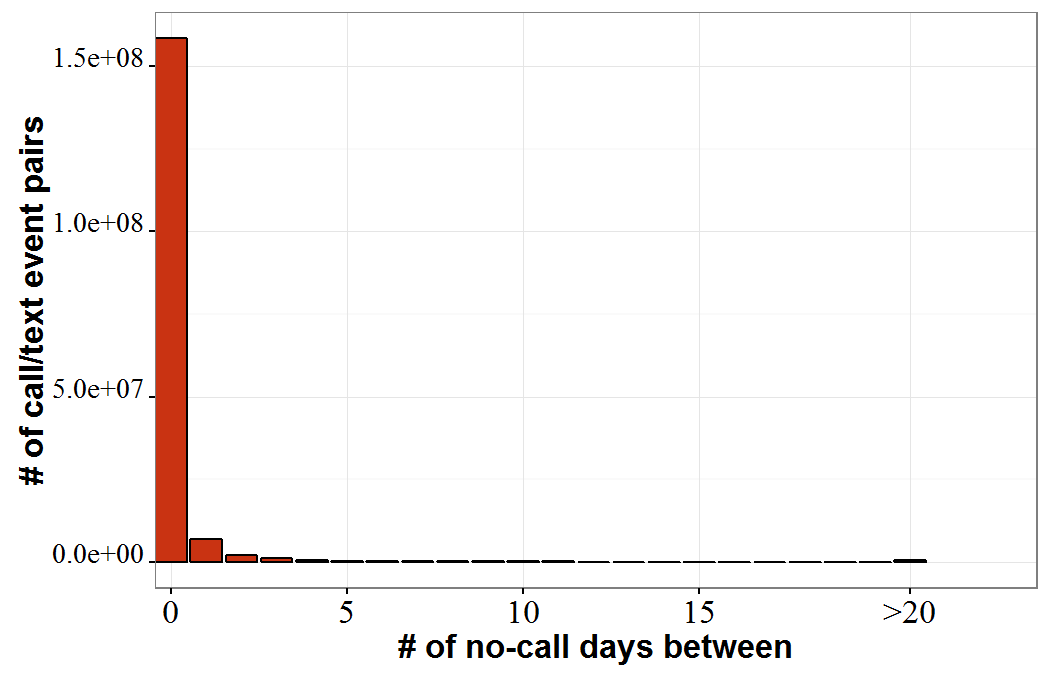


Figure S1. Number of days without calls between call/text events between September and November 2010.

Call/text events may be biased towards occurring during travel, and this bias could affect transition probabilities if some areas are more likely to have travellers than others. Specifically, because we model probability of transitioning between administrative units between call/text events, more frequent call/text events in popular travel areas may make transition probabilities out of these areas artificially low. In areas with few travellers, then, less frequent calls may make transition probabilities out of these areas artificially high. Figure S2 shows the average number of days between call/text events that both happened within the same arrondissement, for each arrondissement, while Figure S3 shows the distribution of numbers of days between events across all arrondissements.


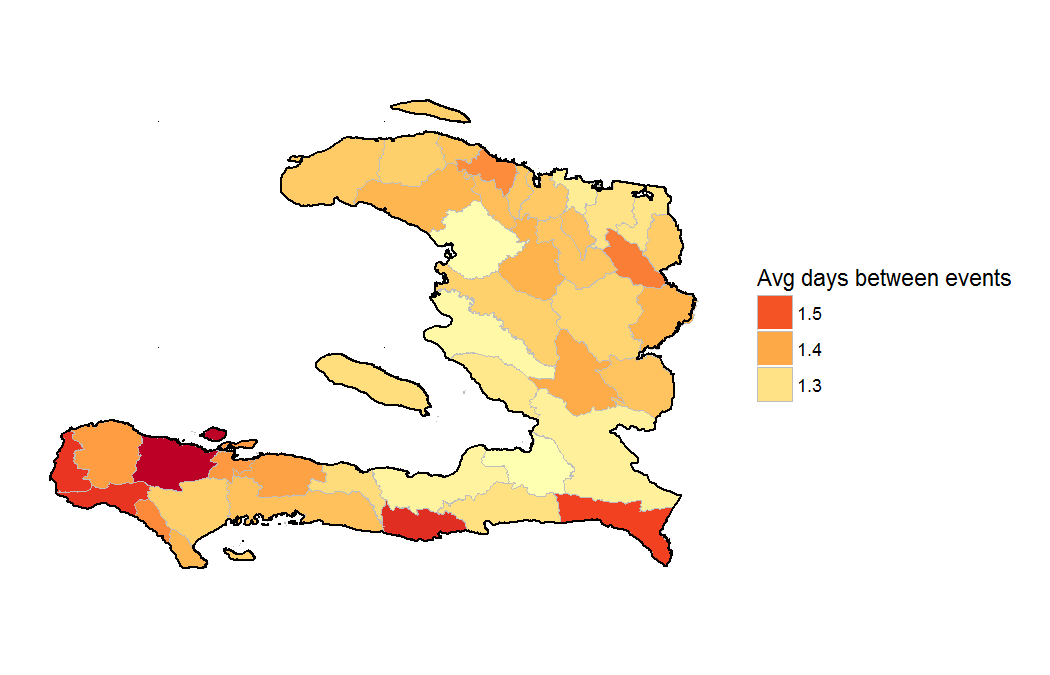


Figure S2. Average time between call/text event pairs that occurred within the same arrondissement, by arrondissement.


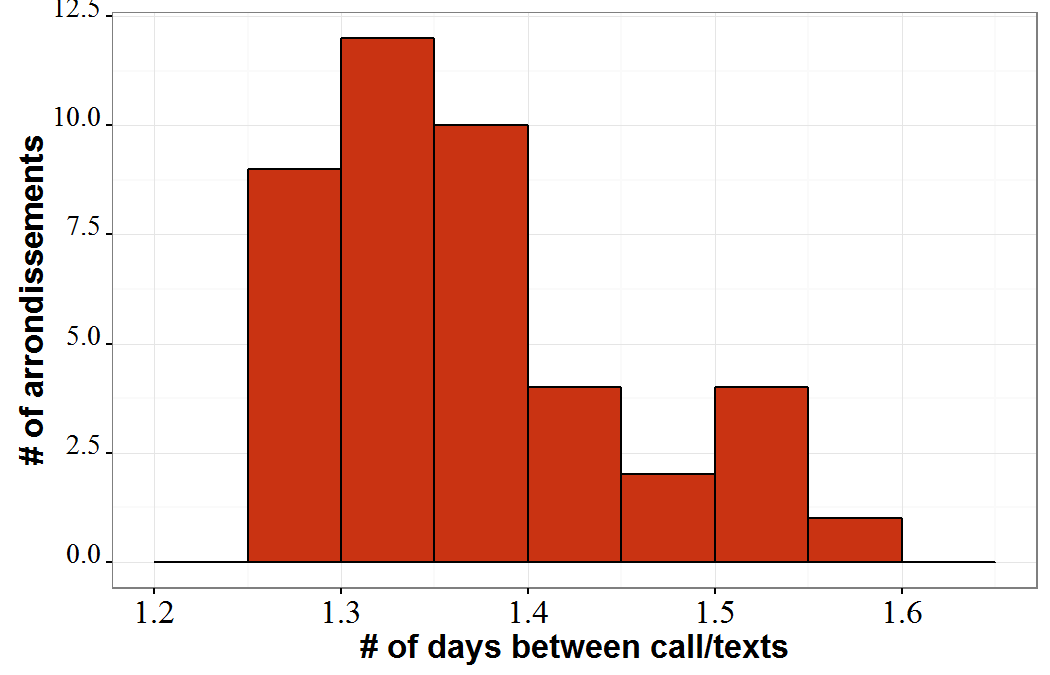


Figure S3. Average time between call/text event pairs that occurred within the same arrondissement, across all arrondissements.

**References**

1. Bengtsson L, Lu X, Thorson A, Garfield R, von Schreeb J. Improved Response to Disasters and Outbreaks by Tracking Population Movements with Mobile Phone Network Data: A Post-Earthquake Geospatial Study in Haiti. PLoS Med. 2011;8:e1001083.

2. Wesolowski A, Eagle N, Noor AM, Snow RW, Buckee CO. The impact of biases in mobile phone ownership on estimates of human mobility. J. R. Soc. Interface. 2013;10:20120986.

3. Tizzoni M, Bajardi P, Decuyper A, Kon Kam King G, Schneider CM, Blondel V, et al. On the Use of Human Mobility Proxies for Modeling Epidemics. PLoS Comput Biol. 2014;10:e1003716.
